# Navigating the ethical complexities of severe and enduring (longstanding) eating disorders: tools for critically reflective practice and collaborative decision-making

**DOI:** 10.1186/s40337-024-01082-0

**Published:** 2024-09-06

**Authors:** Sacha Kendall Jamieson, Jacinta Tan, Kym Piekunka, Shannon Calvert, Stephen Anderson

**Affiliations:** 1 Sydney School of Education and Social Work, The University of Sydney, Sydney, Australia; 2 Imperial College London and My Lighthouse Ltd, London, UK; 3 ResilientSiblings.Com, Port Hueneme, CA USA; 4 Healing Conversations, Perth, WA Australia; 5 NHS Greater Glasgow & Clyde, Glasgow, Scotland

**Keywords:** Severe and enduring eating disorder (SEED), Severe and enduring anorexia nervosa (SE-AN), Longstanding eating disorders, Ethics, Lived experience, Practice guide, Human rights, Cultural safety, Critical reflection

## Abstract

Decisions about the treatment of eating disorders do not occur in a socio-political vacuum. They are shaped by power relations that produce categories of risk and determine who is worthy of care. This impacts who gets access to care and recognition of rights in mental health services. Globally, there are calls for more human rights-based approaches in mental health services to reduce coercion, improve collaborative decision making and enhance community care. Treating individuals with longstanding, Severe and Enduring Eating Disorders (SEED) or Severe and Enduring Anorexia Nervosa (SE-AN) can be particularly problematic when it involves highly controversial issues such as treatment withdrawal and end-of-life decisions and, where legally permissible, medically assisted dying. In this article, we argue that the socio-political context in which clinical decision making occurs must be accounted for in these ethical considerations. This encompasses considerations of how power and resources are distributed, who controls these decisions, who benefits and who is harmed by these decisions, who is excluded from services, and who is marginalised in decision making processes. The article also presents tools for critically reflective practice and collaborative decision-making that can support clinicians in considering power factors in their practice and assisting individuals with longstanding eating disorders, SEED and SE-AN to attain their rights in mental health services.

**Supplementary Information:**

The online version contains supplementary material available at 10.1186/s40337-024-01082-0.

## Abstract (plain language summary)

Navigating the ethical issues involved in the treatment of longstanding eating disorders, Severe and Enduring Eating Disorders (SEED), and Severe and Enduring Anorexia Nervosa (SE-AN) can be difficult for many clinicians. These include highly controversial issues such as treatment withdrawal, end of life issues, and medically assisted dying. In this article, we argue that the socio-political context in which clinical decision making occurs must be accounted for in these ethical considerations. This encompasses considerations of how power and resources are distributed, who controls these decisions, who benefits and who is harmed by these decisions, who is excluded from services, and who is marginalised in decision making processes. The article also presents tools for critically reflective practice and collaborative decision-making that can support clinicians in considering power factors in their practice and assisting individuals with longstanding eating disorders, SEED and SE-AN to attain their rights in health services.

**Supplementary Information:**

The online version contains supplementary material available at 10.1186/s40337-024-01082-0.

## Introduction

Globally, there are calls for more human rights-based approaches in mental health services to reduce coercion, improve collaborative decision making and enhance community care [1, 2]. This article considers the question of how to assist individuals with longstanding eating disorders, Severe and Enduring Eating Disorders (SEED), and Severe and Enduring Anorexia Nervosa (SE-AN) to attain their rights in the context of neoliberal health policy. As an authorship team, we bring different perspectives and experiences to this dialogue. However, we collectively aim to provoke a conversation about ethical decision making for the treatment of longstanding eating disorders, SEED and SE-AN where the socio-political context of these decisions is accounted for. Drawing on knowledge of health systems and their impacts in three neoliberal health policy contexts (Australia, the U.K. and the U.S), we argue that neoliberal politics are relevant to the ethics of treatment of longstanding eating disorders, SEED, and SE-AN. Neoliberal politics are characterised by the valorisation of competition and capital accumulation, with corresponding economic policies of deregulation, marketisation, and privatisation of public health and social care services [3]. Neoliberal politics additionally aim to reinforce capitalist goals by shaping social relations in ways that undermine forms of social solidarity that restrain capital accumulation [3]. These politics can challenge the implementation of human rights-based approaches in health care in terms of reducing coercion, collaborative decision making and enhancing community care. However, clinicians can resist this through critique of the operation and use of power in clinical settings and by utilising practices that shift power to patients and their loved ones. This article presents tools for ethical practice that can support clinicians to pursue this in their work. Whilst this may not impact changes in funding and resourcing in clinical services in the short term, changes in practice within services can influence advocacy and policy reform in the long term.

## Authors’ standpoints/perspectives

Sacha Kendall Jamieson is a social work academic whose research and teaching is focused on the moral-political activity of professional work, anti-carceral practice, and health equity. Sacha has practiced as a social worker in acute adult eating disorders services in Australia, where she had the privilege of advocating for the rights of consumers in legal and clinical processes and contributing to multidisciplinary discussions where critical reflection on professional power was valued.

Jacinta Tan is a retired child and adolescent psychiatrist, clinical academic and medical ethicist. She is an honorary senior lecturer at Imperial College London and based in the United Kingdom. She has spent decades thinking about ethical issues and decision making in eating disorders, but also brings a more recent personal perspective as someone living with significant disability caused by a career-ending chronic illness (Long Covid) for which there are few answers and even fewer solutions for recovery.

Kym Piekunka is the owner of Resilient Sibs, a platform dedicated to highlighting the sibling experience and supporting their journey. Her work is informed by the impact of her sister's severe bulimia nervosa, addictions, and co-occurring illnesses over a span of 15 years. During this period, Kym and her family faced numerous challenges, including inconsistent or denied care, limited family support, and ultimately, her sister’s passing. Although based in the United States, Kym reports that siblings worldwide continue to share similar narratives.

Shannon Calvert is an Australian Lived Experience advisor, drawing from her decades of navigating complex health systems while living with a long-standing eating disorder and complex trauma. Her advocacy focuses on building meaningful partnerships across sectors to enhance the quality of care for both providers and recipients through compassionate, dignified, and trauma-informed treatment and interventions. With extensive experience advising both government and non-government organisations, Shannon specialises in co-designing policies, research, and educational frameworks that prioritise the voices and needs of those with lived experience.

Stephen Anderson is a consultant psychiatrist in eating disorders, working with adults within the National Health Service in Scotland. He has had an interest in medical ethics since medical school and in the legal aspects of treating people with eating disorders in his clinical role. He has had concerns about young people being transferred from adolescent to adult services, already with labels such as ‘treatment resistant’, or ‘severe and enduring’ and follows with interest, and concern, the developing landscape of medical assistance in dying around the world for people with mental disorders.

In recognition of the politics of representation and the power of language to be inclusive or stigmatising, we include the following note on language used in this article.^1^

## Background

Clinicians are empowered by their societies to make important moral as well as clinical judgements, for instance, whether to offer or withhold or impose treatment and how to manage finite medical resources. However, in neoliberal health policy contexts, decisions about the just distribution of scarce medical resources and care are increasingly complex and in conflict with the values held by health professionals [4–6]. Governments have reduced resourcing for public services and health inequity has increased [7–10]. Within publicly funded health services, overriding value is placed on efficiency, exemplified in performance indicators to determine service resourcing and the proliferation of standardised ‘techno-rationalist’ tools and frameworks to simplify clinical assessment and monitor and measure clinical activities [11]. Eligibility criteria for care has become stricter [8, 12, 13]. Risk discourse is pervasive and may challenge ethical practice if bureaucratic risks are prioritised over assisting patients with the ‘risks’ they are facing [14]. Although there can be no standardised measures for ‘recovery’, as this is a complex person-centred concept [2, 15, 16], individuals may be denied care if it isn’t possible for clinicians to guarantee measurable benefit from treatment [17, 18]. Individuals, their loved ones, and communities are held responsible for preventing acute illness without a corresponding increase in resourcing of community mental health services or other services and initiatives addressing social determinants of mental health [19–21]. This produces a pernicious cycle where the demand for acute care increases alongside decreasing resources for prevention and rehabilitation, placing unsustainable pressure on clinical services and the community [22].

Within clinical services, body weight and medical risk are the primary measures for determining access to treatment, thus individuals with higher weights or who do not meet thresholds of acuity, may be denied care until they are ‘sick enough’ [17, 23]. Risk discourse is pervasive in mental health services [14, 24]. Decisions about acuity, severity, urgency, capacity, and what is reasonable regarding limitations of rights are imbued with considerations about risk. Everyday conversations between clinicians include: risk of harm due to non-intervention, risk of harm if intervention is delayed, risk of harm due to intervention, patient risk to self, patient risk to others, risk of death, risk to quality of life and dignity. Clinicians may be required to use standardised risk assessment tools, despite critiques that they are reductive, biased in their selectiveness of risks considered, underpinned by Western cultural assumptions, stigmatising of the individual, and unable to predict risk [14, 25–27].

Underpinned by the notion that risk is predictable, risk assessment tools problematically place responsibility for the management of risk onto clinicians. In these circumstances, clinicians may be inclined to prioritise their own risks [28] or to have a lower threshold for the use of coercion and involuntary treatment [29]. Individuals who return to health services after receiving treatment, as is often the case with longstanding eating disorders, may be labelled as ‘treatment resistant’, having ‘complex needs’, or ‘non-compliant’, conferring a deviant moral status. This can result in withholding of treatment on the basis that the service cannot meet the individuals’ needs, or, if they are admitted for treatment, they may receive involuntary or punitive ‘care’ [30, 31]. Clinicians can be reluctant or averse to treating patients with these labels due to the uncertainty around outcomes, corresponding perceived risk to the clinician’s expert status, and the ramifications for how they will be perceived and treated by colleagues [18, 32].

Standardised risk assessment tools can also constrain ethical practice, encouraging a particular kind of professionalism based on expertise in risk management rather than advocacy or critical reflection on the use of discretionary power, for example [32, 36]. This is significant in terms of the application of rights-based approaches aiming to reduce coercion, improve collaborative decision making and enhance community care. Clinicians hold the position of power in the patient relationship to determine how rights are interpreted and whether coercion or compulsory treatment will be used. Clinicians may draw on knowledge about the patient’s values, priorities, and support network to reduce coercion, enhance collaboration and promote community care. However, the clinicians’ thoughts and feelings over time about their effectiveness, the value of treatment, the futility of treatment, and ‘risk’ could also lead to the use of discretionary power that devalues the patient’s voice, knowledge, values, and priorities, limiting their rights to non-coercive treatment, participation in decision making and community-based care [37–39].

We argue that implementation of a rights-based approach therefore requires tools for countering risk discourse and critical reflection on how rights are interpreted and applied in ethical decision making. This is ethically imperative in neoliberal health policy contexts when considered against the following impacts and harms:

1. Risk discourse obscures that systems produce longstanding, severe and enduring illnesses and endanger people’s lives through the withholding of care.
2. Risk discourse can generate an unhelpful ‘us and them’ dynamic between clinicians and individuals who ‘don’t respond to treatment’.
3. When resources are scarce, the unhelpful ‘us and them’ dynamic between people with lived experience can deepen, with those conferred as ‘deviant’ (and their loved ones) being stigmatised as unworthy of resources or a burden on the system.
4. The focus on managing patient risk diverts attention from services and individual clinicians as potential risks, permitting a lack of scrutiny and evaluation of how systems and clinical interventions can be harmful, thwarting service improvement.
5. Social solidarity between clinicians, the individuals they work with, and those individuals' loved ones is systemically denied, undermining collective approaches to reform.
6. For many clinicians, this makes the treatment environment increasingly fraught. A clinician’s tolerance for this may be contingent on whether and to what extent they experience ‘ethical stress’ [40–42]. This will depend on complexities such as power dynamics and support within their clinical team, personal beliefs/views/values, and the clinician’s position of privilege in society in terms of their own identity and advantage within current socio- political arrangements.

The practice of ‘critical reflection’ recognises that where professionals claim to respect the dignity and autonomy of individuals, do no harm, and address social injustices, they must be accountable for their use of power. Critical reflection involves actively recognising that your own social and cultural background, emotions, assumptions, and interpretations shape your construction of a situation and ideas about the best response. A critically reflective approach to practice values multiple perspectives and dialogical processes that privilege voices that have previously been silenced or marginalised. Moreover, it involves critiquing one’s own practice, acknowledging that people can experience harm in systems, sometimes as a result of policy and sometimes as a result of direct practice [43, 44].

Tools that encourage critical reflection can support clinicians in working with uncertainty (countering risk discourse) and asserting their role as advocates. This is particularly relevant in eating disorder treatment, as clinicians are further challenged by matters of nomenclature and evidence, as discussed in the next section. We then present five tools to assist clinicians in this endeavour.

### The ethics of diagnostic categorisation

The terms ‘severe and enduring eating disorder (SEED)’ and ‘severe and enduring anorexia nervosa (SE-AN)’ are widely accepted and applied within eating disorder treatment services; yet they are not well defined [15, 45, 46]. With this, a new risk has arisen: people who attract that label may find themselves marginalised, stigmatised and unworthy, as hard-pressed clinicians with limited resources prioritise treating other patients. Triaging of scarce resources may be disguised as futility. The label of SEED/SE-AN could become a self-fulfilling prophecy, with all involved giving up hope or expectation of recovery [47]. It is important to note, however, that SEED/SE-AN itself encompasses a wide range of illness statuses, and only a small proportion have a high or imminent risk of dying.

We suggest that the literature frequently conflates various types of definitions when they refer to people who have more enduring eating disorders. We propose the following categories (among potential others):

- A first and most substantial group consists of individuals with longstanding eating disorders. Despite not fully recovering after several years, they maintain stable lives. This group is frequently identified in population research and may not currently receive specialised treatment. It is important to acknowledge that stability and support needs change over time. [46–51].
- A second group includes individuals with severe and enduring eating disorders whose severity primarily impacts their daily functioning. They may periodically require medical or psychiatric treatment and intermittent therapy [52, 53].
- A third subgroup consists of those with severe eating disorders significantly affecting their medical and psychiatric health, sometimes posing a life threat. These individuals often require intensive treatment and support [47].
- Finally, there is a fourth subgroup of individuals with severe and enduring eating disorders experiencing acutely challenging situations that impact their quality of life, evoking despair in themselves, their loved ones, support networks, and treating clinicians [37, 39, 54].

Distinguishing these categories is crucial because SEED/SE-AN labels do NOT apply to everyone with enduring eating disorders. Needs and experiences can vary significantly, even with a long-standing eating disorder. Some individuals find support and stability outside of specialised treatment, while others require ongoing care throughout their journey. This highlights the importance of individualised care and the potential risks of misinterpretation associated with unclear definitions. It is important to maintain an ongoing, open dialogue about treatment options, even during periods of stability.

**Fig. 1:**
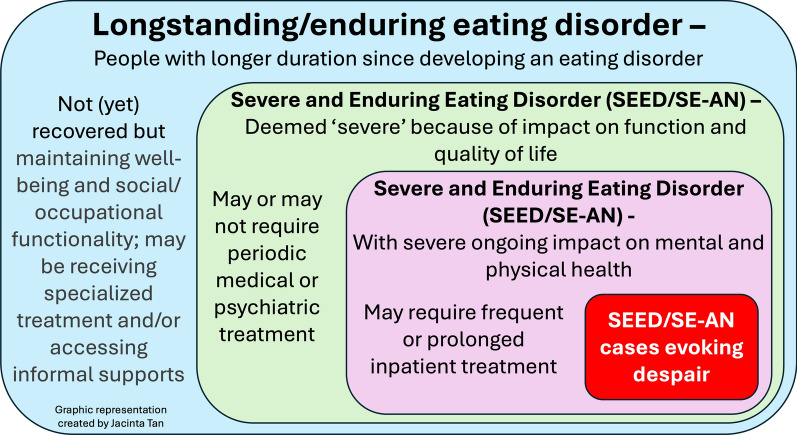
Different and conceptually distinct subgroups related to SEED/SE-AN

While we don’t have statistics on the numbers of people in these subcategories, it seems the final category, which evokes the most despair, is likely the least common, yet generates the most controversy. This focus risks overlooking the needs of other SEED/SE-AN subgroups who face significant challenges without being considered acutely life-threatening. These categories are conceptually depicted in Fig. 1 as subcategories, each within the preceding one.

Our best evidenced treatments significantly help only around 50% of adults with anorexia nervosa [55–57]. Approximately 20% of people with anorexia nervosa develop an enduring illness [58, 59]. There is evidence that people with enduring forms of illness may benefit from modifications of standard treatment [60, 61]. Predictors of poor treatment outcomes are known, but these are for grouped outcomes, so it is impossible to predict which individual may recover; furthermore, recovery is possible even after more than 20 years of illness [62, 63].

Geppert et al. discussing ‘futility’ suggest that ‘unless the motivation and minimum ability to nourish oneself remains, people with lived experience with SEED who “throw in the towel” can be thought of as entering end stage disease’ [64]. They admit, however, that there are no clear prognostic signs and symptoms of end stage illness that render further treatment futile as there are in metastatic cancers and other diseases. It is suggested that some people with lived experience with an enduring, unremitting illness who are unable to maintain a minimally agreed-upon weight and nutritional status may request a model of care focused on symptomatic relief [54].

Gaudiani and colleagues have recently presented three cases and an argument supporting their definition of ‘terminal anorexia nervosa’ [65]. Note that this would be applicable only to a specific group within the previously mentioned "cases evoking despair" category. They discuss the use of medical aid in dying (MAID) which is available in some States in the USA and some other countries such as Canada, the Netherlands and Belgium. This proposal has caused significant unease and anger among some patient and carer groups and fellow clinicians, with some arguing strongly against the idea of defining terminal anorexia nervosa, and others disputing the possibility of developing such criteria where resources drive decisions rather than the uncertainties of the illness, on the basis of human rights and dignity [22, 30, 66, 67].

### Polemic debate as dangerous

This controversy has created polemic and entrenched positions regarding policy or approach. The situations being discussed are necessarily emotive and troubling situations, which are always complex in unique and varying ways for each individual, caregiver and family. Polemic academic debate by its dichotomous nature can mask these complexities, diminishing the dialogue involved in making decisions about how rights should be interpreted and applied. It also risks dismissing voices and impacting the quality and nature of these difficult journeys for people with lived experience and their loved ones. Further, this complexity may make it difficult for clinicians, particularly those who are more junior, to be open about the challenges they are facing. In a neoliberal political context, diminishing the complexity of work could also lead to less resourcing.

While cases of SEED/SE-AN that evoke despair in clinicians deserve attention, a narrow focus on these cases can obscure the broader inequities in access to resources and care for people with SEED/SE-AN. This risks diverting crucial resources and attention away from a significant proportion of individuals with SEED/SE-AN who grapple with long-term challenges but may not present as the most acute cases.

These individuals' rights and needs are often disregarded or overshadowed by more dramatic cases, potentially silencing their voices and those of their loved ones in decision making processes. This marginalization can contribute to a sense of "pervasive unworthiness" [47] and further stigmatize all patients with longstanding eating disorders and SEED/SE-AN, regardless of their current risk to life or health. It is crucial to remember that suicide rates among people with eating disorders are alarmingly high [68], highlighting the overall severity of, and despair surrounding, these conditions.

Polarised debate also problematically reinforces an ideology that ethics is about agreeing on rules for practice, as if all people with lived experience with the same diagnostic label were the same; that clinicians are experts of their patient’s experiences, and risk is predictable and easily quantified; that treatment always provides safety and benefit; and that there aren’t power dynamics within multidisciplinary teams and healthcare systems that undermine collaborative decision making and silence dissenting voices [69].

## Tools for practice

In this section of the article, we present five tools designed to support clinicians in critically reflective, collaborative decision making processes where multiplicity and diversity of perspectives is actively elicited, respected and valued. The tools aim to promote open and curious practice that genuinely seeks to empower and hear from people with lived experience and their loved ones, to be informed and led by their values and priorities.

### Tool 1: adopting a values-based approach

Values-based mental health is an approach intended to sit alongside evidence-based mental health [70–72]. In it, the values of the people with lived experience and their loved ones are elicited and prioritised alongside those of the clinicians and then integrated with best evidence and other issues to enable consensual decision making [73–76]. These are illustrated in Text Box 1 [77].

**Text Box 1:**
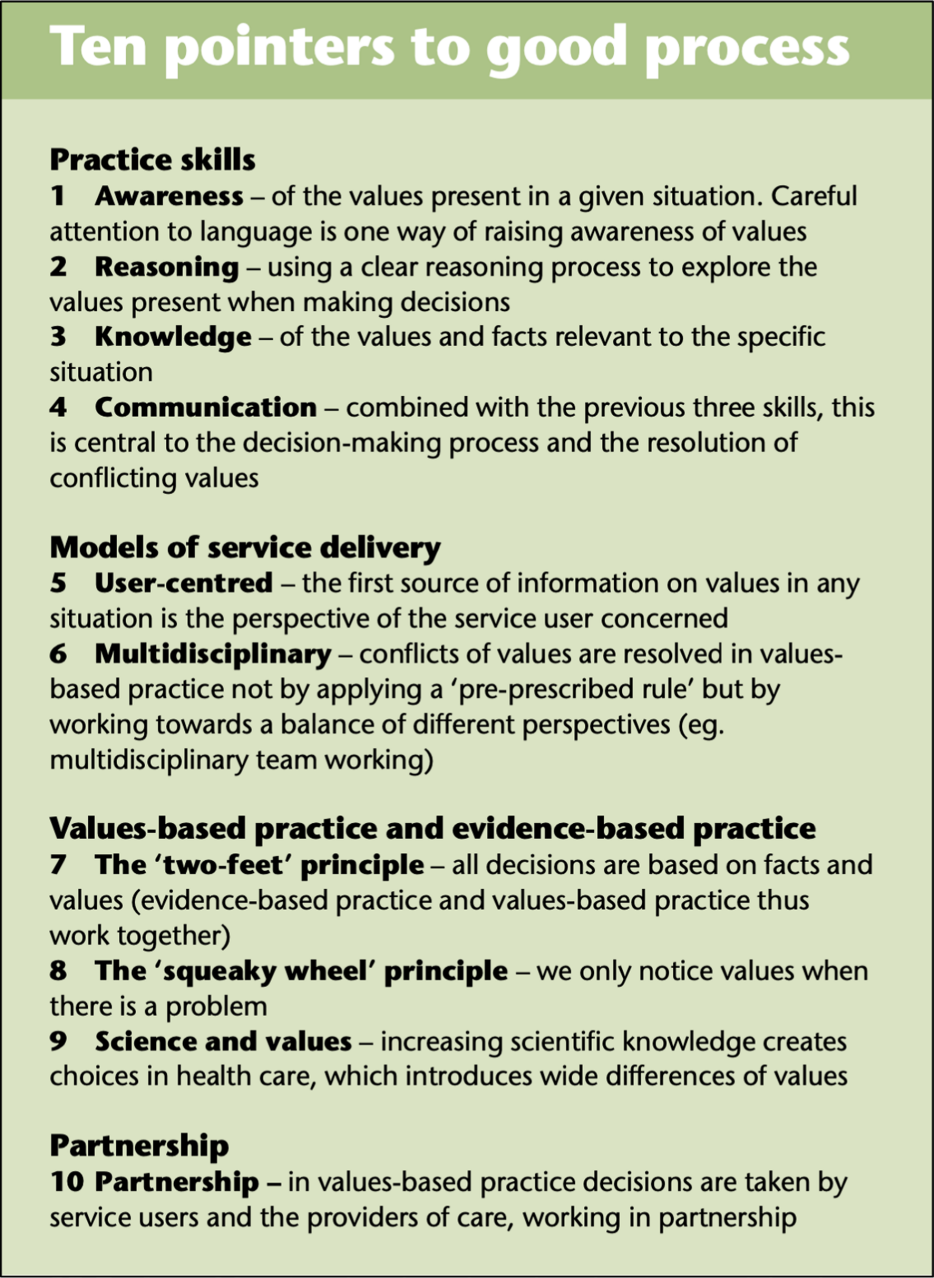
Ten pointers to good process in Values Based Practice

### Tool 2: Adopting critical reflection within clinical teams

**Fig. 2:**
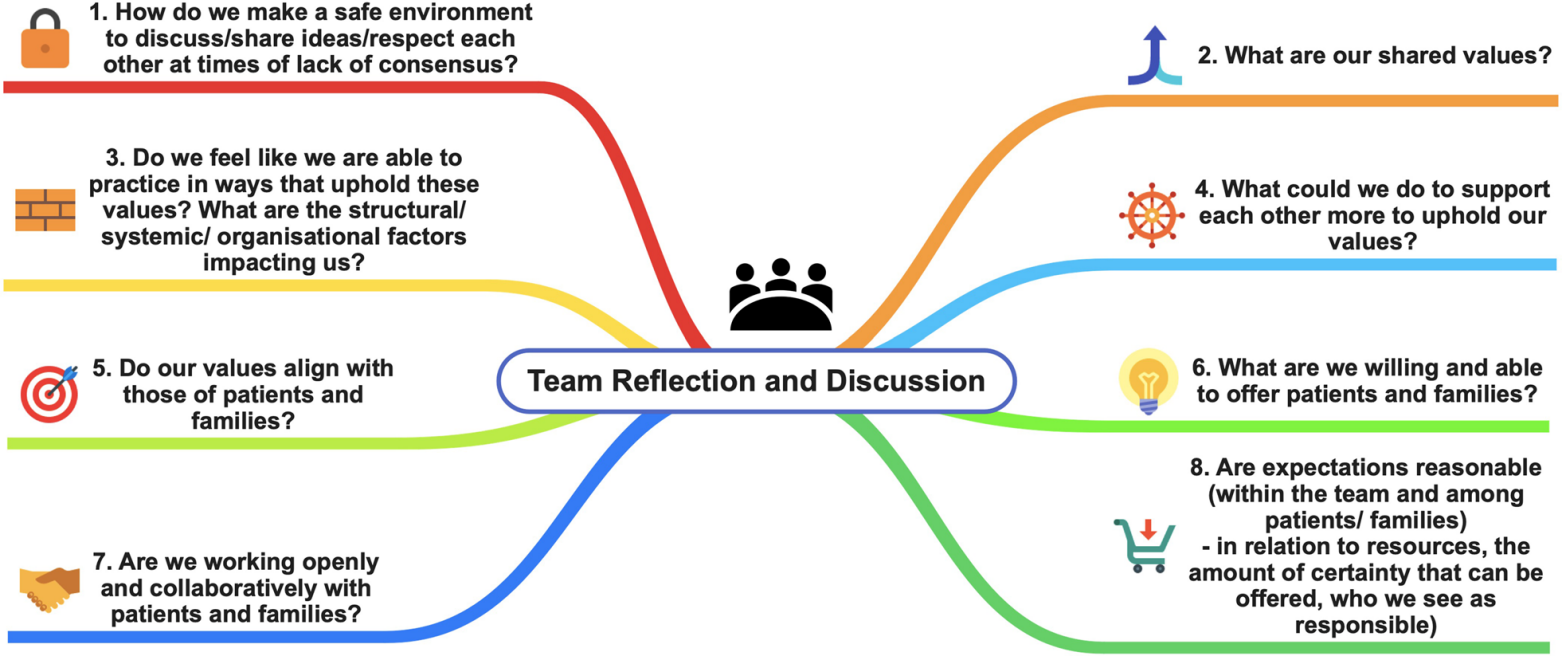
Team reflection and discussion [79]

Because of the power imbalance inherent in delivering treatment it is important that clinicians develop and maintain as a priority a critically reflective culture, as the bedrock upon which to engage in respectful approaches to people with lived experience and their loved ones. The following Fig. 2 provides a set of 8 discussion questions that may support treating teams in this process, including multi-agency discussions or discussions between clinical teams, for example between primary care and specialist services [78, 79].

### Tool 3: Employing cultural safety principles with people with lived experience and their loved ones

Cultural safety is a practice approach for addressing systemic racism in health care [80]. Developed by First Nations scholar and nurse, Irihapeti Ramsden, the approach begins by acknowledging that First Nations people in colonial contexts are systemically denied their rights in all systems, including health. This is connected to a lack of understanding of different cultural concepts of health, a lack of understanding of the ongoing health impacts of colonisation, and racism. This makes health services inequitable and dangerous due to stereotyping, inadequate assessment, misdiagnosis, denial of care, prejudicial treatment and unjust deaths [81]. Fundamental to the approach is ‘cultural humility’. This concept invites clinicians to question how their social and cultural identity and privilege in society is shaping their assumptions, interpretations, and judgements and to be accountable for their power in an ongoing way. Enacting this approach will depend on the context and vary depending on the patient. However, guiding practice principles are recognising that racialised communities have experienced significant harm in health systems and commitment and action to not replicate this harm *and* make services ‘safe’. Clinicians can do this by showing humility, positioning themself as a learner by listening to people with lived experience and their loved ones and centring their values, perspectives and priorities in all interpretations and planning [82].

We have drawn on the cultural safety literature to consider how to support clinicians in assisting people with lived experience with longstanding eating disorders, SEED and SE-AN to attain their rights in health systems. This is about learning from people with lived experience and their loved ones about what rights-based approaches to treatment and support look like for them, seeking to understand their experience, values and priorities [79]. Such an approach is particularly important for people with lived experience with longstanding eating disorders, SEED/SE-AN and their loved ones, as they have endured years of multiple treatments, and become experts in their own illness and treatment experiences.

**Fig. 3:**
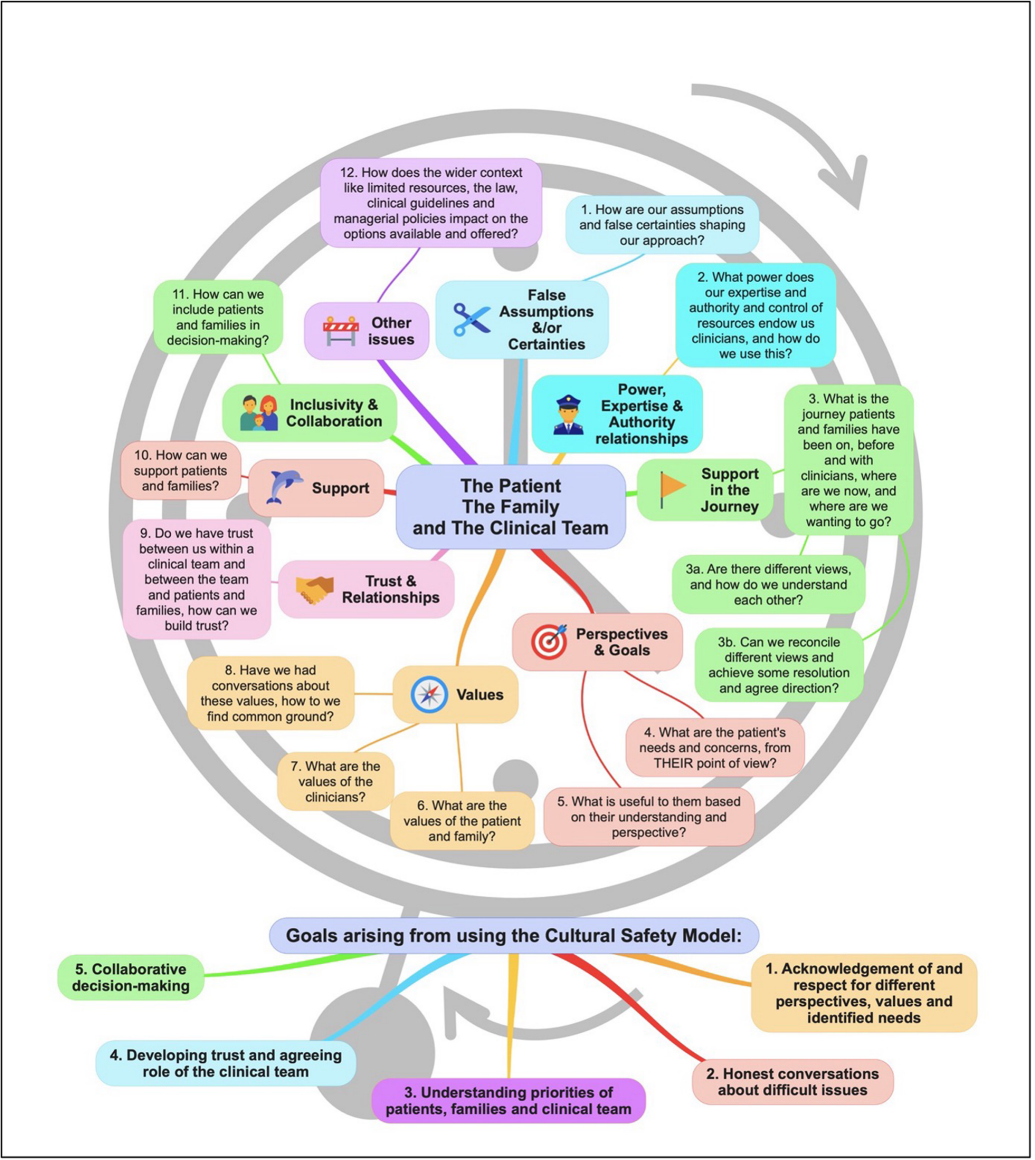
Visual representation of the Cultural Safety Model for eating disorders [79]

Figure 3 and Text Box 2 offer tools to support the ethical practice considerations discussed above. Figure 3 provides a set of questions for teams to collaboratively explore interdisciplinary perspectives, concerns, and uncertainties, building a foundation of safety and respect before interacting with individuals and their loved ones. Text Box 2 presents an ethical framework in the form of questions to guide clinicians, individuals, and their support networks in engaging in open dialogue and reaching shared treatment decisions. Importantly, we do not assume a specific age for the individual or a traditional family structure. The term 'family' encompasses parents, partners, other relatives, and any trusted members of the individual's community who they wish to involve in their care.

**Text Box 2:**
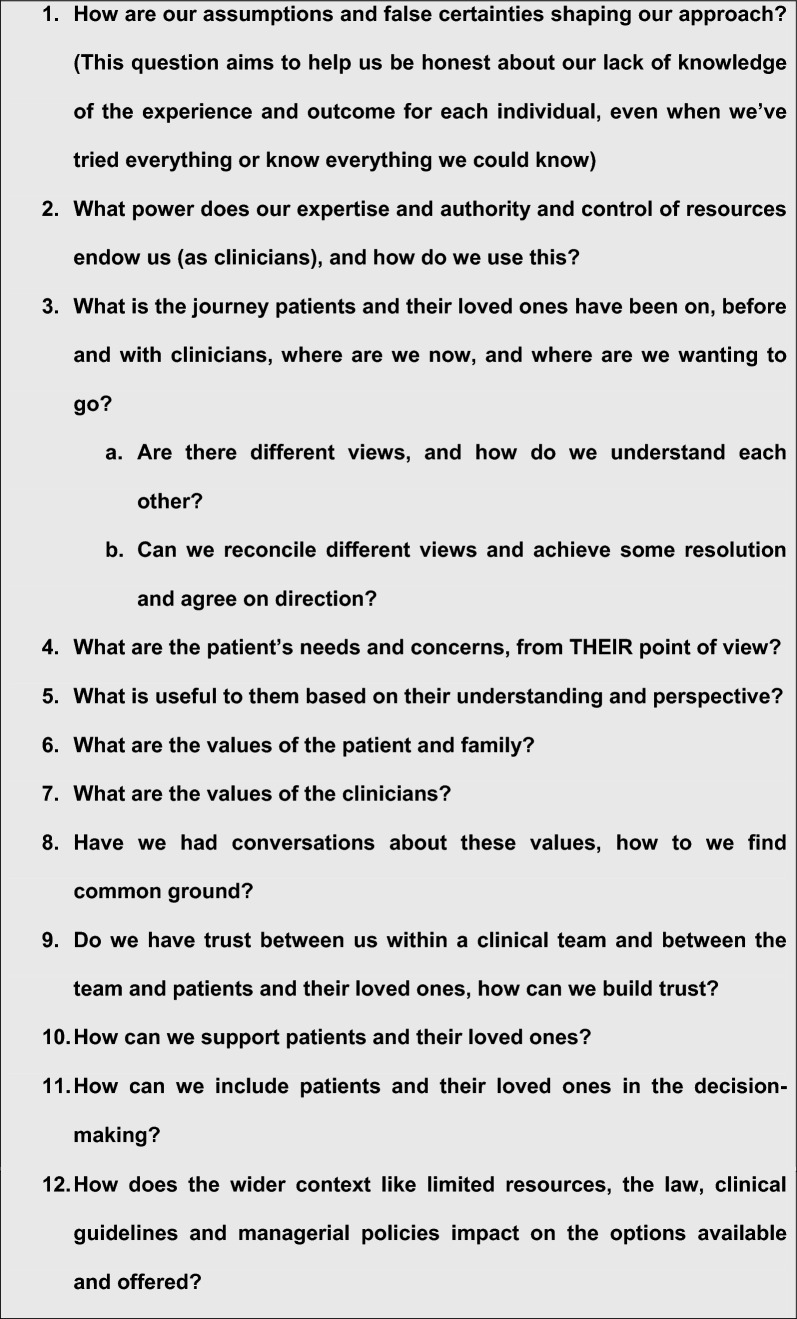
Questions to use in the Cultural Safety Framework

In the visual representation, the questions on the framework in Text Box 2 are depicted as hours on a clockface with the intention that the discussions work round the clockface. The overarching goals are depicted as the pendulum of the clock (see Fig. 3).

### Tool 4: utilising the four framework approach

**Fig. 4:**
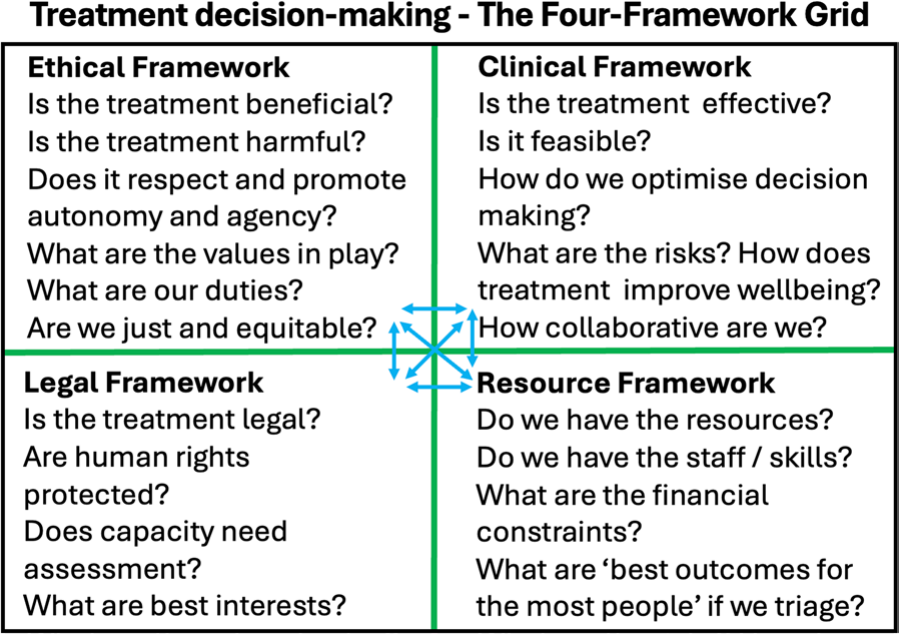
The Four Framework Grid [83]

The Four Frameworks Approach [83] is a framework that clinicians can use to disentangle and comprehend the different constraints and pressures they face. For example, are resource constraints leading to clinicians concluding that certain people with lived experience cannot be helped, because they are employing utilitarian principles of ‘triaging’ services to deliver the most quality adjusted life years (QALYs) for the most people with lived experience? Or are clinicians simply ‘firefighting’, responding by treating the most dangerously unwell patients at the expense of treating the much larger group who may be less unwell and more easily helped? Or is fear of breaking the law or transgressing on human rights leading to clinicians being risk averse? The Four Framework Grid (see Fig. 4) does not offer any solutions to clinicians because all clinical decisions must be made on an individual basis. Instead, the hope is that with reflective practice and open discussion (see Tools 1, 2 and 3), employing the grid to disentangle and distinguish the kinds of questions needing discussion will enable critical understanding of the influence of various drivers of decisions. This promotes open communication with patients and their loved ones about resource constraints and treatment options. Identifying sources of moral distress can also motivate clinicians, people with lived experience (patient/consumer and carer/family), advocacy groups and organisations to lobby for change.

### Tool 5: the decisions involved in low weight and enduring medically-risky anorexia nervosa and other eating disorders framework (DILEMA)

Much as it is painful for all, there is a sub-group of people with SEED/SE-AN for whom the continuation of ‘treatment as usual’ can become unhelpful or harmful. This particularly applies to those ‘evoking despair’ where active treatment must be repeatedly delivered compulsorily or with restraint and loss of liberty as inpatients. This can lead to considerable suffering and erosion of dignity. There have been several such cases heard in the Court of Protection in England and Wales, where the general outcome has been that these people with lived experience lack the capacity to make decisions about their treatment because of the severe and entrenched nature of their disorder; yet the judges have often not found it in their best interests to continue to be compulsorily treated [84]. In other jurisdictions, individuals with lived experience have been allowed to avail themselves to Medically Assisted Dying [85, 86]. In such and similar cases, there could come a time when there should be honest consideration of whether a temporary pausing of active or aggressive treatment or even consideration of how to help people to be comfortable and maintain dignity when facing the prospect or possibility of decline and death [87].

**Fig. 5:**
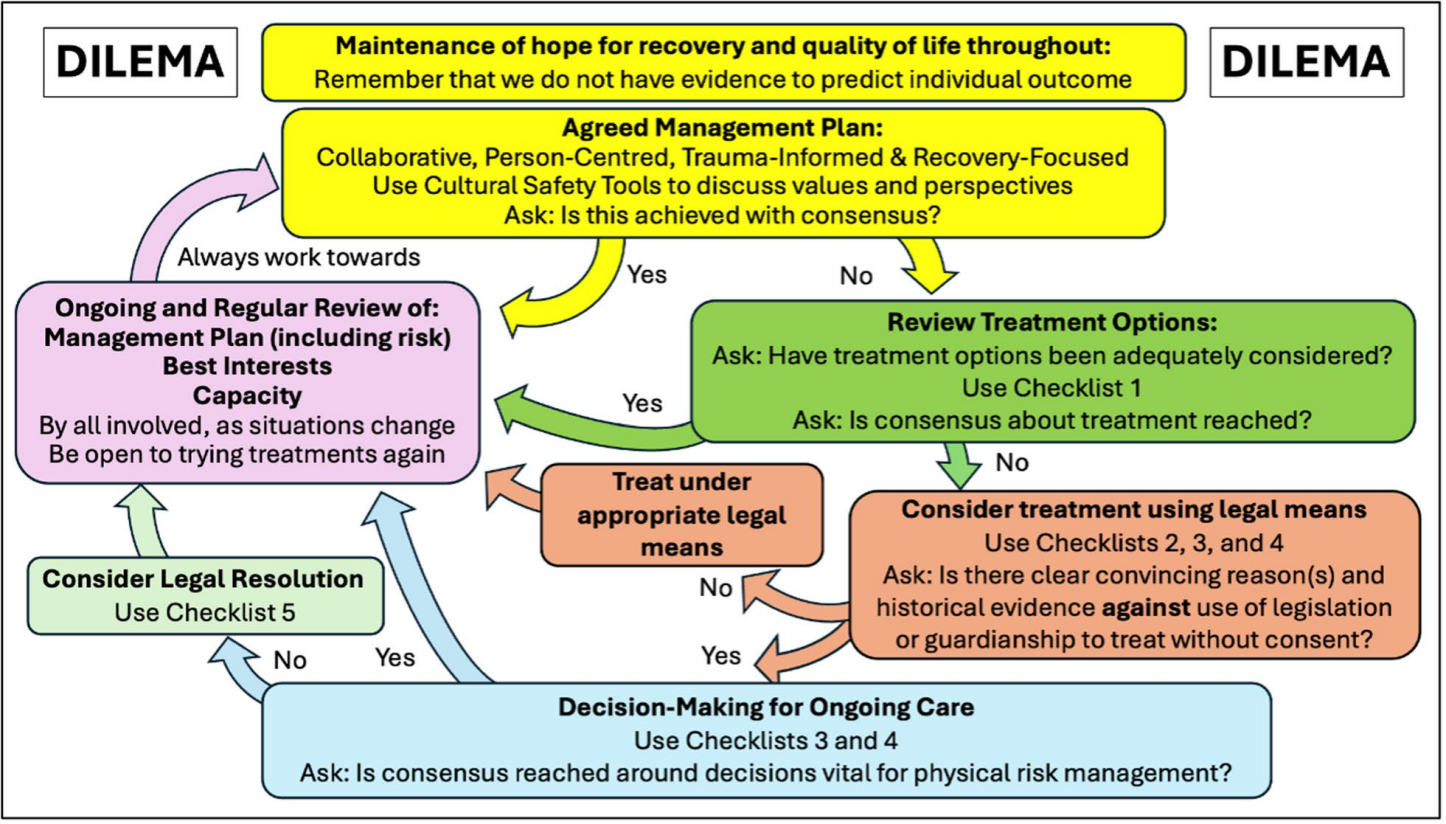
The DILEMA Framework

The ‘Decisions Involved in Low weight and Enduring Medically-risky Anorexia Nervosa and other eating disorders framework’ (DILEMA) has been developed by four eating disorder psychiatrists, including a medical ethicist, to support clinicians in addressing these difficult decisions. The framework seeks consensus in all areas of consideration and is compatible with the Values Based Practice approach, the Cultural Safety Tool, and the Four Frameworks Grid. Clinicians are encouraged to employ all these tools when considering each step within the DILEMA Framework (see Fig. 5).

As discussed in this article, there can be significant difficulties in the use of language in this very complex area. Working on DILEMA over more than three years, it became increasingly clear that wording and language of any framework will be viewed and interpreted through the lens of the reader, and not necessarily what was intended by the authors. It takes a format that is deliberately circular in nature and agnostic about decisions made or outcomes, because these must remain individual to each person and context. The word ‘framework’ is deliberate. This is not a pathway, a set of ‘to do’ checklists, a solution, or a prescriptive guideline. It is intended to be collaborative and to maintain hope for recovery and quality of life, and the checklists are intended to ensure systematic consideration of various issues and questions. It is further intended to support clinicians, people with lived experience and their loved ones and support networks, to allow all of them to be collectively involved in these incredibly complex and often stressful and distressing situations. This systematic framework allows for ongoing, iterative and collaborative reviews of management plans which are both flexible and responsive to changes in various factors. It is also expected that the goals and treatment approaches would shift depending on the journeys of the clinicians, patients and loved ones, as well as in response to new opportunities and treatment options.

These situations where people with lived experience are at serious risk but there is uncertainty about the way forward can lead to emotionally-driven and inadequately thought-through decision making. For example, on one hand, clinicians may feel obliged to repeatedly detain and compulsorily and restrictively treat patients when there is no evidence of benefit because managers are concerned about outcomes, or a fear of being judged as neglecting their duty of care by their peers, governing bodies or medical coroners. This dynamic has been seen in some of the Court of Protection cases in England and Wales [84]. On the other hand, clinicians may be drawn into the patient and/or the family’s sense of hopelessness and futility about ongoing treatment options. The reverse can also occur, that people with lived experience and their loved ones could be impacted by clinicians’ sense of despair or hopelessness. In any of these situations, DILEMA can provide a framework to allow all involved to step back from the heat of the situation and consider, with the support of systematic questions set out in checklists, what options may be helpful to consider. Text Box 3 lays out what DILEMA is, and what it is not.

**Text Box 3:**
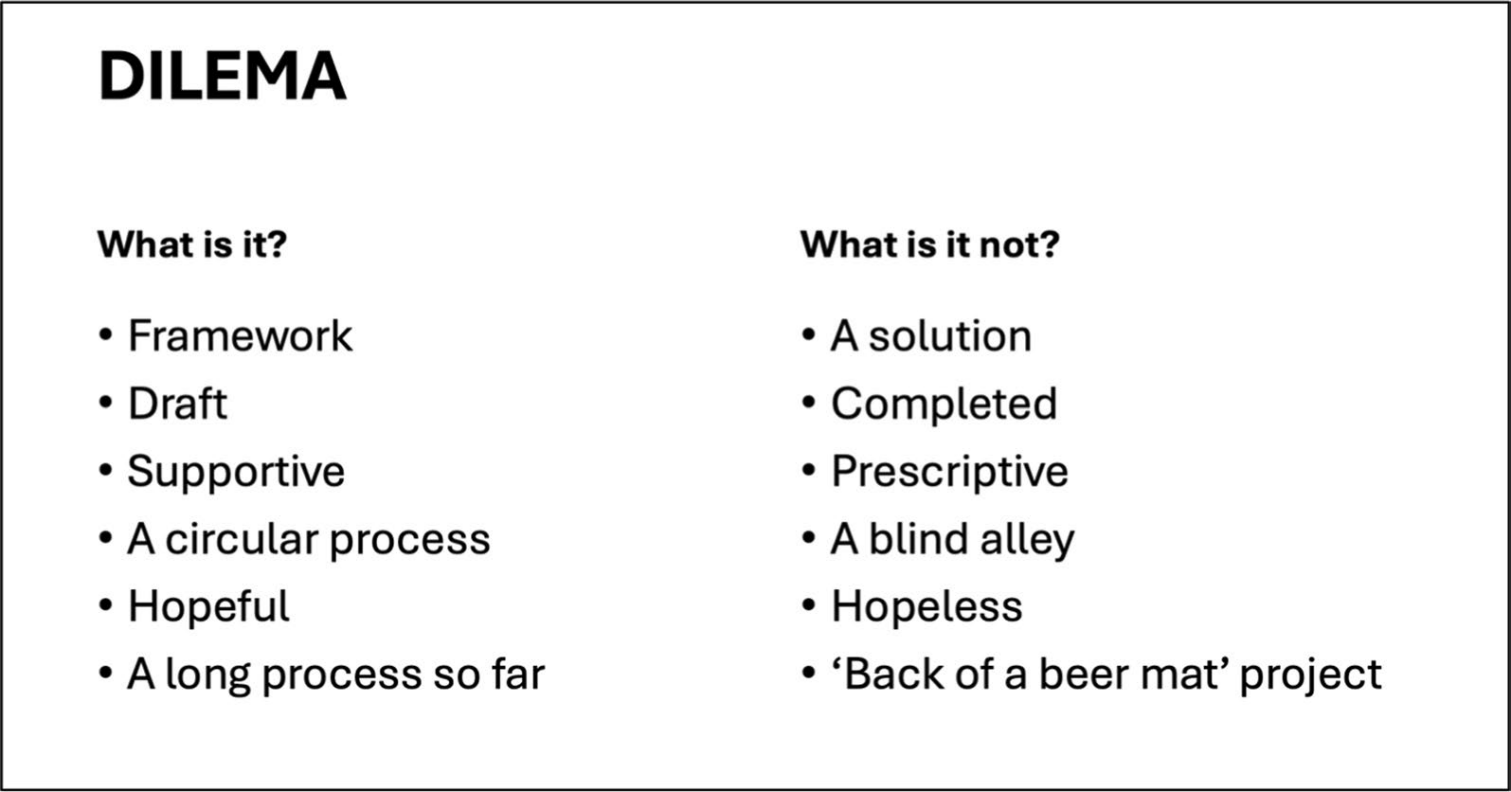
The Decisions Involved in Low weight and Enduring Medically-risky Anorexia Nervosa and other eating disorders framework (DILEMA)

Integrated into the DILEMA framework is a series of Checklists, provided in Text Boxes 4, 5, 6, 7 and 8. It is recommended that clinicians, people with lived experience and their loved ones go through these checklists and answer each question in turn according to the particular views, experiences and circumstances that are relevant, in order to systematically consider and discuss the various issues involved and arrive at consensus or, alternatively, a plan acceptable to all bearing in mind respectful ‘dissensus’ of differences of position and opinion.

**Text Box 4:** DILEMA Checklist 1: Treatment Options

1	There have been attempts to treat over a long period of time (we suggest at least 7 years of intensive treatment in high quality adult specialist eating disorder services), ideally with continuity of care by staff who know and understand the patient well and have good therapeutic relationships with the patient
2	When considering treatment or inpatient admissions, consider whether a treatment or admission which has been historically unsuccessful may now be helpful or justified because of changes in context, circumstances, or perspectives of clinical teams, patients and loved ones which have occurred. Be particularly careful of making assumptions that the outcomes of a treatment or admission which occurred a long time ago would predict outcome now
3	Consider whether it is justified to attempt to restore healthy weight or stable medical status. If it is agreed that this is not in best interests of the patient, this should be justified by historical attempts to restore healthy weight or stable medical status in a specialist inpatient setting, as well as lack of any change in context, circumstances, perspectives or patient wishes
4	Consider whether all appropriate treatment options have been explored, including under the appropriate legal means as needed. If it is agreed that particular treatment options are not in best interests of the patient, this should be justified by historical attempts to implement them in the relevant specialist treatment setting, as well as lack of any change in context, circumstances, perspectives or patient wishes
5	Inpatient treatment has ideally taken place in more than one specialist service. If this has only taken place in one specialist unit or with one specialist team, careful consideration should be given to whether it may help the patient to experience treatment in a different setting where a different approach and new therapeutic relationships can be tried
6	There have been attempts to restore and maintain a negotiated (minimally) safe weight in a specialist inpatient or community setting, using a rehabilitation/recovery approach
7	All options for treatment in the community have been considered
8	Consideration has been given to specialist eating disorder rehabilitation units or other supportive accommodation or treatment settings
9	There has been access to a range of evidence based psychological therapies of adequate duration, both during inpatient treatment, and as ongoing care post discharge
10	Other recognised approaches (not necessarily listed in current clinical guidelines or best practice documents) have been equally considered
11	Revisiting of previous attempted treatments has been considered
12	Comorbidity has been adequately considered and treated, with collaborative working across services
13	As far as possible, the patient has been involved in review and decision making
14	As far as possible, their loved ones, or appropriate advocates, have been involved in review and decision making
15	Second opinion, ideally independent, has been considered

**Text Box 5:** DILEMA Checklist 2: Consider using legal means (usually mental health or guardianship) to treat compulsorily without consent

1	It is expected that there has been a recent attempt to treat compulsorily using legal means appropriate according to jurisdiction
2	It is expected that patients would usually have been detained using legal means on several occasions in a specialist eating disorder unit or, if such a unit is unavailable, to another inpatient setting under the care of an eating disorder consultant and specialist team
3	Consider what it was like last time, or at previous times the patient was treated using legal means? Was it effective or helpful overall? Did the use of legal means allow the patient to engage more effectively with treatment, for example giving the patient the ability to relinquish control, or supporting the patient to make good decisions about their care in spite of underlying anorexic drive? If it was not helpful overall, what were the reasons, and would it be possible to address these in order to justify another attempt at using legal means?
4	Did the benefits of the use of using legal means in the long-term outweigh the detrimental effects, such as the effect on the therapeutic relationship, distress to the patient (possibly from the use of enforced weight regain through nasogastric feeding and/or restraint), sense of coercion, removal of autonomy, effect on the wider family, trauma related sequalae, impact on life opportunities, or time away from home and family?
5	Consider whether any factors suggest that using legal means to provide treatment will work differently this time, including different patient and carer and clinician opinions from before, or any new or different interventions available, that can be delivered using legal means?
6	Based on a detailed knowledge of the patient, and the views of the family, and others consulted, consider whether the level of distress from using legal means is justifiable and balanced in terms of the likely gains (short-term, medium term and long-term)
7	Should a second opinion be considered to help with this decision?

**Text Box 6:** DILEMA Checklist 3: Decision-making for ongoing care

1	Gather all opinions for example patient, advocate, family, other health professionals or other advisory bodies (for example, Mental Welfare Commission (Scotland))
2	Consider obtaining advice from an Ethicist or a Clinical Ethical Committee, if available
3	Consider meetings to discuss treatment options. Several meetings may be needed. Thought should be given to who needs to be present at these meetings
4	Consider the patient’s capacity to make decisions for each decision in question. They may have capacity to make some decisions even if they lack capacity to make others. Other decisions where patients lack capacity may require best interest principles
5	Explicitly discuss risks, including risks of death
6	Attempt to reach a consensus on a management plan including crisis planning, contingency plans and overall medical care
7	Consider a fresh approach to treatment, initially for a trial period with frequent reviews, with the goal to reduce suffering through comfort care alongside support for patients to manage their eating disorders, for example nutritional support and medical monitoring. This should ideally be an interdisciplinary medical caregiving approach aimed at optimizing the quality of life and mitigating suffering among patients and their loved ones who face the problems associated with the illness. This should be through prevention and relief of suffering by means of early identification and impeccable assessment and treatment of pain and any other problems, including physical, psychosocial and spiritual. Shifting the goals can also provide relief and a space to reconsider goals together, especially when there has been protracted conflict or use of compulsion
8	This is a dynamic and iterative process to continue to revisit the plan and goals as circumstances change
9	Consider a Second Opinion to support decision-making
10	If there is difficulty reaching a consensus, consider involving senior colleagues, or clinical managerial colleagues (maybe within Eating Disorder services or may be from other mental health specialities), or an independent clinician to facilitate the meeting, for example, a senior colleague, a clinical manager, or an experienced expert colleague from another service, for example, the clinical lead for the regional provider collaborative in NHS England

**Text Box 7:** DILEMA Checklist 4: Capacity Assessment

*Note: This is an abbreviated version of the original Checklist, which is provided in Appendix 1*
We suggest using the following format, which is based on the MacCAT-T instrument [86–88]. Any full assessment of capacity requires careful reference to the mental capacity legal definition and criteria in each particular location of clinical practice. It further requires careful inquiry into aspects unique to eating disorders which may affect decision-making capacity, such as changes in values related to the eating disorder, changes in authenticity and personal identity, and difficulty with appreciating the facts of the eating disorder apply to them due to cognitive and perceptual distortions [81, 89–92] Optimise setting for a capacity assessment If the patient is stressed or anxious, provide support 1. Assess ability to understand and retain information A. Information about the illness or disorder that the patient is assessed as having and the risks related to this illness; and B. Information about the benefits and risks of at least two treatment options, which may include the option of refusing treatment, in order to offer a choice for the decision 2. Assess ability to use information 3. Assess appreciation of information and facts of the decision 4. Assess presence of (internal) compulsion 5. Assess for changes in values due to the eating disorder 6. Assess for changes in identity due to the disorder 7. Assess for depressive features, loss of hope and affective elements 8. Look for other specific difficulties 9. Global clinical assessment 10. Consider asking for a second opinion assessment of capacity if uncertain

**Text Box 8:** DILEMA Checklist 5: Legal resolution

1	Consider obtaining advice or involvement from your health organisation’s legal team
2	Consider legal mediation
3	Consider advice or involvement from your personal and professional indemnity organisation

## Conclusions

The treatment of people who have longstanding eating disorders, SEED or SE-AN can be both clinically difficult and morally fraught [64, 89]. There can be difficulties from a multitude of sources—lack of good evidence to guide individual treatment choices, difficulties balancing best interests with diminishing quality of life and loss of dignity and agency, resource constraints, legal constraints, management of risk, management of expectations and distress both in clinicians and people with lived experience and their loved ones, to name but a few. All the while, there is also a particular danger of feelings of frustration, fear, hopelessness, rejection, abandonment and unworthiness driving decisions and pushing people into unhelpful polarised positions [15].

The five tools we offer may help clinicians in identifying and then collaboratively discussing different relevant factors that may quickly become conflated or entangled. They are also designed to promote ongoing learning in critical reflection and self-awareness, along with a commitment to inclusion and respect for diverse perspectives and values when trying to achieve consensus between people with lived experience, loved ones, and clinicians within teams and between teams. Fundamentally, these tools are about taking action to redress power imbalances and prevent marginalisation, stigmatisation and other forms of system harm by collaborating with people with lived experience and their loved ones to determine the ‘right’ framings and decisions for them.

## Supplementary Information

Additional file 1.

## Data Availability

No datasets were generated or analysed during the current study.

## Abbreviations

SEED: Severe and enduring eating disorders
SE-AN: Severe and enduring anorexia nervosa
VBP: Values based practice
DILEMA: Decisions involved in low weight and enduring medically-risky anorexia nervosa and other eating disorders framework (DILEMA)
QALYs: Quality adjusted life years
MAID: Medical assistance in dying

## Footnotes

1 In this article, we will use the term ‘clinicians’ to refer to all healthcare professionals involved in the treatment of eating disorders, regardless of their specific discipline, We will use the terms ‘people’, ‘person’, ‘individual’ to describe those with lived experience of an eating disorder. The term ‘patient’ will be used specifically when discussing their involvement in the healthcare system. We will use the term ‘ loved ones ‘ to encompass the broad range of support networks in a person’s life. This can include parents, partners, adult children, friends, chosen family, and anyone who provides support and care for the person with lived experience.
