# Supplementary material for: Navigating the ethical complexities of severe and enduring (longstanding) eating disorders: tools for critically reflective practice and collaborative decision-making
Source: J Eat Disord. 2024 Sep 6;12:134. doi: 10.1186/s40337-024-01082-0 (PMC11378593; doi:10.1186/s40337-024-01082-0)
Supplement: Supplementary file 1 — Additional file 1. [file 40337_2024_1082_MOESM1_ESM.docx]

**Appendix 1: Checklist 4 – Capacity Assessment (full version)**

Note: While formal capacity assessments are crucial for documenting reasons to override treatment refusal, we recommend an ongoing, dynamic process. This includes considering a patient's potential difficulties in agreeing to and cooperating with treatment. Through open dialogue with patients and their loved ones, capacity assessment can facilitate transparent, collaborative decision-making. This patient-centered approach allows clinicians to tailor treatment plans while addressing any challenges the patient might face. For more formal assessments, careful documentation of every step is essential.

We recommend using the following format, based on the MacCAT-T instrument, for a comprehensive capacity assessment in individuals with eating disorders. The MacCAT-T is clinically validated for psychiatric settings and is particularly valuable due to its systematic approach and detailed documentation.

Setting:

Any capacity assessment, formal or informal, requires suitable conditions to ensure optimal engagement. Avoid rushed or stressful environments, as these may underestimate a patient's decision-making abilities. An ideal setting includes:

a) A calm and quiet space

b) Privacy without distractions or interruptions

c) Sufficient time for thorough exploration of the issue

d) An environment of low expressed emotion and minimal pressure

e) Ideally, an assessor familiar with and trusted by the patient

If the patient feels stressed or anxious, consider the presence of a supportive person (e.g., staff member, loved one) who understands they should not interfere with the assessment process.

**1. Assess ability to understand and retain information**

The MacCAT-T is a clinically validated method of assessing capacity in psychiatric patients. It is particularly helpful here in terms of how it requires to examiner to systematically go through all the information and check understanding and retention.

Basically, using this format comprises of careful provision of specific information (which is pre-determined and fully documented by the clinician assessor) in manageable chunks, and at each stage asking the patient to repeat in their own words what was said to them. Requiring the patient to repeat back in their own words enables the assessor to check comprehension and retention of the information. Where there is incomplete understanding or retention, the assessor should carefully explain the information again, in more simplified form or smaller chunks as needed, and then ask the patient to repeat back again in their own words.

There are two key parts to the information needed:

1. Information about the illness or disorder that the patient is assessed as having and the risks related to this illness; and

In order to offer a choice for the decision, information about the benefits and risks of at least two treatment options, which may include the option of refusing treatment, should be provided.

The aim is to enable a full understanding of the information required for the decision in question. Assessors should only assess the patient as failing to understand or retain information if it becomes evident after several attempts. Any failure to understand and retain information immediately puts the capacity to make that decision in question. Please note that people with lived experience with eating disorders rarely fail in this section.

**2. Assess ability to use information**

This is also part of the MacCAT-T instrument. Ability to use the information can be assessed during the course of the entire discussion. It is specifically elicited by asking the patient to generate possible consequences for each treatment option and to compare the treatment options in terms of likely benefits and risks. It is also elicited by asking the patient to explain for his/her reasons for the decision. As the patient explains his/her reasoning it should become evident whether the patient is able to use the information provided.

**3. Assess appreciation of information and facts of the decision**

Appreciation, not seen in UK legislation but found in Grisso and Appelbaum’s definition of competence (1,2), is the ability to apply the illness and treatment information to oneself. This can be a problem in eating disorders, for example, a patient may say, “I understand that’s the definition of an eating disorder, I understand I have those features, and I understand eating disorders need treatment; but I do NOT have an eating disorder and therefore I do not need treatment”. Or, they may say “I understand eating disorders may be fatal but those facts don’t apply to me, I would not die from it.” These failures of appreciation, which are similar to but more specific than loss of insight, would clearly affect capacity to make decisions about treatment for an eating disorder. It is important to try to ascertain any other acceptable reasons or rationale for apparent failure of appreciation, for example a longstanding alternative world view preceding the illness that mental disorders are merely a construct built upon medical tyranny may be accepted as a reason that someone might not accept that they have a mental disorder.

**4. Assess presence of (internal) compulsion**

Look for compulsions (or obsessions) that may prevent the patient from acting on the basis of his/her understanding or even desires. The Code of Practice of the Mental Capacity Act 2005 for England and Wales gives an example that patients with anorexia nervosa may be unable to ‘use and weigh’ treatment information as part of the decision-making process: “4.22 For example, a person with the eating disorder anorexia nervosa may understand information about the consequences of not eating. But their compulsion not to eat might be too strong for them to ignore.” (3)

**5. Assess for changes in values due to the eating disorder**

It is part of the core criteria of anorexia nervosa that a person should either have a fear of fatness, or an overvaluing or pursuit of thinness. This dread of fatness and overvaluing of thinness, found in many eating disorders, means that being thin or losing weight or not gaining weight may be disproportionately highly valued by sufferers, in some cases even valued above life itself. This disproportionate value can drive some patients to decide, even after they have weighed up the options according to those values, not to have treatment because they would rather die than gain weight. It is important to assess whether a difference is values is due to the disorder, or due to other legitimate reasons such as culture, ethnicity, alternative lifestyles/world views or personal or religious beliefs.

**6. Assess for changes in identity due to the disorder**

One of the characteristics of eating disorders is that they can be egosyntonic disorders, that is, experienced as part of the self and also as consistent with one’s own values, such that people may not be able to distinguish the drives of the disorder from their own wishes or desires (4). Further, many people with eating disorders become ill as adolescents, and may as adults have little or no sense of who they would be without the disorder. This intertwining of the disorder with the sense of self can make it difficult to decide to have treatment in order to recover from it; for example, patients may be either unable to envisage a self without eating disorders and therefore feel unwilling or unable to choose to recover, or fear what they might become if free of their eating disorder.

1. **Assess for depressive features, loss of hope and affective elements**

Eating disorders heavily impact emotions and mood, with high rates of co-occurring depression. Careful assessment of depressive features and suicidality is crucial. It is vital not to dismiss the absence of overt suicidal thoughts. Patients might express a passive desire for death linked to their eating disorder (e.g., wanting to die thin). Assess for hopelessness and a lack of hope or vision for recovery, as these significantly affect a person's decision-making process and perception of risk. Some individuals with lived experience, driven by hopelessness and worthlessness, may not prioritize the preservation of their lives. Others might experience such intense suffering that death becomes a form of escape, sometimes even actively sought.

Charland and colleagues further argue that beyond comorbid depressive disorder, anorexia nervosa itself may have clear affective components, fitting Ribot’s historical conception of a ‘passion’ in its very nature (5). These 'passion' components, including a fixed focus, intrinsic motivational force, and strong attachment, may drive individuals with lived experience towards specific treatment options and significantly impact their decision-making.

**8. Other specific difficulties**

People with lived experience can have other specific features of their illness which may affect capacity and reasons people make decisions. Some might experience a critical internal voice urging them to reject food and treatment, creating a sense of compulsion. Others may feel they would face negative consequences from their eating disorder if they sought recovery. Still others may harbor contradictory beliefs about treatment, desiring it yet feeling incapable of choosing it due to the illness. Exploring an individual's motivations and reasoning is crucial for understanding these factors that can hinder their capacity.

**9. Global clinical assessment**

The final summary of the capacity assessment should be laid out in according to the legal criteria of capacity according to where you practise, but can be informed by all the points above.

The bottom line is that a binary global clinical judgement of ‘has capacity/does not have capacity’ may needed for a specific decision at the time of assessment. The graver the decision the more careful the assessment of capacity should be. For example, if a request for assisted dying is in question, then the assessment of capacity should be very careful and considered, and ideally involve independent assessors or second opinions.

**Final comments:**

While a binary clinical judgment of capacity might be legally necessary for specific decisions, this assessment should be nuanced. A lack of capacity in one area does not equate to a global lack of capacity. Regardless of formal assessment, respect the individual with lived experience by actively involving them in the decision-making process.

The framework of capacity also helps identify a patient's wishes and need for support. Compulsory treatment, or decisions by the clinicians to act in best interests, can be experienced as supportive (6). Collaborate with the patient and their support network to help them access their values and goals, potentially fostering their ability to overcome the influence of their eating disorder. If uncertainty remains about capacity, seek a second opinion.

1. **Consider asking for a second opinion assessment of capacity if uncertain.**

**References**

1. Grisso T, Appelbaum PS. Assessing Competence to Consent to Treatment. Oxford: Oxford University Press; 1998. 224 p.

2. Appelbaum PS, Grisso T. The MacArthur treatment competence study. I: Mental illness and competence to consent to treatment. Law Hum Behav. 1995;19(2):105–26.

3. Office of the Public Guardian. Mental Capacity Act Code of Practice [Internet]. London; 2016. Available from: https://assets.publishing.service.gov.uk/government/uploads/system/uploads/attachment_data/file/497253/Mental-capacity-act-code-of-practice.pdf

4. Tan J, Hope T, Stewart A. Anorexia nervosa and personal identity: The accounts of patients and their parents. Int J Law Psychiatry. 2003;26(5):533–48.

5. Charland LC, Hope T, Stewart A, Tan J. Anorexia Nervosa As A Passion. Philosophy, Psychiatry, & Psychology. 2012;(4):353–65.

6. Tan J, Hope T, Stewart A, Fitzpatrick R. Control and compulsory treatment in anorexia nervosa: The views of patients and parents. Int J Law Psychiatry. 2003;26(6):627–45.
